# Gene polymorphisms associated with non-alcoholic fatty liver disease and coronary artery disease: a concise review

**DOI:** 10.1186/s12944-016-0221-8

**Published:** 2016-03-10

**Authors:** Xiao-Lin Li, Jian-Qing Sui, Lin-Lin Lu, Nan-Nan Zhang, Xin Xu, Quan-Yong Dong, Yong-Ning Xin, Shi-Ying Xuan

**Affiliations:** Department of Gastroenterology, Qingdao Municipal Hospital, Dalian Medical University, Qingdao, 266011 China; Department of Gastroenterology, Qingdao Municipal Hospital, Qingdao, 266011 China; Digestive Disease Key Laboratory of Qingdao, Qingdao, 266071 China; Central Laboratories, Qingdao Municipal Hospital, Qingdao, 266071 China

**Keywords:** Non-alcoholic fatty liver disease, Coronary artery disease, Gene pathogenesis, Genetic polymorphisms

## Abstract

Non-alcoholic fatty liver disease (NAFLD) is a common chronic liver disease which represents a wide spectrum of hepatic damage. Several studies have reported that NAFLD is a strong independent risk factor for coronary artery disease (CAD). And patients with NAFLD are at higher risk and suggested undergoperiodic cardiovascular risk assessment. Cardiovascular disease (CVD) is responsible for the main cause of death in patients with NAFLD, and is mostly influenced by genetic factors. Both NAFLD and CAD are heterogeneous disease. Common pathways involved in the pathogenesis of NAFLD and CAD includes insulin resistance (IR), atherogenic dyslipidemia, subclinical inflammation, oxidative stress, *etc*. Genomic characteristics of these two diseases have been widely studied, further research about the association of these two diseases draws attention. The gene polymorphisms of adiponectin-encoding gene (*ADIPOQ*), leptin receptor (*LEPR*), apolipoprotein C3 (*APOC3*), peroxisome proliferator-activated receptors (*PPAR*), sterol regulatory elementbinding proteins (*SREBP*), transmembrane 6 superfamily member 2 (*TM6SF2*), microsomal triglyceride transfer protein (*MTTP*), tumor necrosis factors-alpha (*TNF-α*) and manganese superoxide dismutase (*MnSOD*) have been reported to be related to NAFLD and CAD. In this review, we aimed to provide an overview of recent insights into the genetic basis of NAFLD and CAD.

## Background

Non-alcoholic fatty liver disease (NAFLD) is recognized as a major public health problem in both developing and developed countries. It is the most common form of liver disease, and represents a wide spectrum of hepatic damage ranging from simple steatosis and non-alcoholic steatohepatitis (NASH) to advanced fibrosis and cirrhosis. NAFLD is recognized as the hepatic manifestation of metabolic syndrome (MS), which major components are insulin resistance (IR), atherogenic dyslipidemia, abdominal obesity, and hypertension [1]. MS is a lethal endocrinopathy involving a chain of systemic disorders such as abdominal obesity, glucose intolerance or diabetes mellitus (DM), dyslipidemia, hypertension and CAD. Nowadays many epidemiological and clinical studies are focused on the association between NAFLD and CAD [2, 3]. Several studies have reported that NAFLD was a strong independent risk factor for CAD in different racial group and with different research methods [4–6]. Meanwhile, a series of studies showed that in NAFLD patients, CAD was the leading cause of death, among the common causes of death after all of the hepatic and extra hepatic malignancy combined [7, 8]. Occurrences of these two diseases are both the result of interactions between multiple genetic variations and environmental factors although a full understanding of pathogenesis has not yet been elucidated. Genetic polymorphisms are reported to influence predisposition of individuals to NAFLD and CAD [9, 10]. Moreover, recent progresses in genomics provide new knowledge of both normal and disease states for precision medicine and systems therapeutics. Therefore, this review aim to summarize the recent advances of correlated genes and the promoting mechanism for NAFLD and CAD, and to assess the possible implication of these polymorphisms on disease morbidity and severity in those patients. It will aid in the development of genes based therapeutic strategies to combat NAFLD and CAD, and could lead to more effective precision medicine to identify the correct strategies for each individual in order to maximize therapeutic effect and minimize the occurrence of adverse reactions.

## Adiponectin-encoding gene (*ADIPOQ*)

Adiponectin-encoding gene, *ADIPOQ*, located on chromosome 3q27, encoding adiponectin protein which is an adipocyte-derived hormone with anti-atherogenic, anti-diabetic and anti-inflammatory properties. In liver, adiponectin protein attenuates insulin resistance by increasing insulin sensitivity. Besides, adiponectin increases endothelial nitric oxide (NO) secretion and inhibits monocyte adhesion and smooth muscle cell proliferation in the vascular wall [11, 12]. In NAFLD patients, adiponectin systemic levels is decreased [13]. *Adiponectin rs266729* (−11377C/G) polymorphism might be a candidate gene, which determines the susceptibility to NAFLD [14]. A study in Indian patients showed an association of two functional polymorphisms, *rs266729* and *rs2241766* (+45 T/G) of *ADIPOQ* with the presence and severity of NAFLD, the presence of G allele at position −11377 correlated with necro-inflammatory grade and at position +45 resulted in reduced plasma adiponectin levels in patients suggesting the functional relevance of these polymorphisms in NAFLD pathogenesis and progression [15]. G allele of *rs266729* is associated with hypoadiponectinemia, and low serum adiponectin level may precipitate liver steatosis in patients with type 2 diabetes, therefore gene polymorphism of adiponectin*rs266729* is associated with the risk of NAFLD [16]. *ADIPOQ* expression decreases significantly in epicardial adipose tissue and paracardial adipose tissue in MS patients with CAD [17]. Foucan L. et al*.* (2010) showed that in Afro-Caribbean patients with type 2 diabetes, *rs2241766* was associated with CAD under a dominant model [18]. A meta-analysis showed that the associations between *rs266729*, *rs2241766* and *rs1501299* (+276G > T) in the *ADIPOQ* and CVD were significant but weak. The *rs2241766* G allele and *rs266729* G allele increase risk of CVD, while the *rs1501299* T allele decreases [19]. *ADIPOQ rs1501299* is association with CAD, and the SNP has different gender dependent effect on adiponectin levels and the lipoprotein metabolism, considering the detrimental effect that the rare homozygous genotype (TT) was associated with higher levels of total cholesterol (TC) and low density lipoprotein cholesterol (LDL-C) compared to common homozygous genotypes (GG) and heterozygous, and the polymorphism influence on the levels of biochemical markers was independent of adiponectin circulating level [20, 21].

## Leptin receptor gene (*LEPR*)

Leptin exerts its physiological action through the leptin receptor (LepR) which is encoded by a single gene on the chromosome 1p31, leptin receptor gene (*LEPR*). The polymorphism of *LEPR* 3057G > A (*rs1805096*) probably contributes to the onset of NAFLD by regulating lipid metabolism and affecting insulin sensitivity [22]. Lys656Asn (*rs8179183*) polymorphism of *LEPR* is associated with insulin resistance and glucose levels in patients with NAFLD [23]. *LEPR* polymorphism *rs1137101*, which is responsible for a Gln223Arg change at the protein level is associated with TC, high-density lipoprotein cholesterol (HDL-C) and LDL-C levels in Japanese men [24]. Polymorphisms of *LEPR* Gln223Arg (G/G) is the risk factors in NAFLD, and the significant interactions between genetic polymorphisms of Gln223Arg added the risk of NAFLD [25]. Gln223Arg polymorphism in the *LEPR* is associated with an increased risk of familial combined hyperlipidemia which is associated with premature CVD mediated by low HDL-C [26]. *LEPR rs1137100* is associated with increased risk of NAFLD and NASH. And it was also associated with simple steatosis and NASH without significant fibrosis [27]. A Japanese study in obese children showed a significantly lower total and LDL cholesterol in subjects with GG genotype [28]. A single nucleotide polymorphism *rs1137100* of *LEPR* results in Lys109Arg change at the protein level and this has been shown to associate with several risk factors for CVD such as body mass index (BMI), systolic blood pressure (SBP), impaired glucose tolerance and insulin sensitivity [29–31]. In Japanese obese children, the individual with Arg109 is association with serum lipids [28]. Carriers of the Arg109Arg genotype or some other polymorphism linked to it seem to be protective from cardiovascular events as well as from total mortality independently of the traditional CAD risk factors [32].

## Apolipoprotein C3 gene (*APOC3*)

Apolipoprotein C3 gene (*APOC3*) is located on chromosome 11q23. Apolipoprotein C3 protein is mainly synthesized in the liver and to some extent in the intestine, and it is a component of triglyceride (TG)-rich lipoproteins and HDL. *APOC3* gene is involved in transport and clearance of chylomicron remnants, and very-low-density lipoprotein (VLDL) and HDL from the bloodstream [33, 34]. A prospective case–control study in the southern Han Chinese population showed that subjects carrying the C allele (TC or CC) of apolipoprotein gene developed insulin resistance (IR) more commonly, and it was in agreement with that found in the Indian population [35]. The polymorphisms −482 C/T (*rs2854117*) and −455 T/C (*rs2854116*) in *APOC3* were associated with NAFLD and IR [35]. The -455 T > C conversion is located in a putative insulin-response element of *APOC3*, which is associated with plasma TG levels [36–38]. A prospective case–control study involving 300 NAFLD patients and 300 healthy controls indicated that *APOC3 rs2854116* genetic variations involved in the susceptibility to develop NAFLD, IR, hypertriglyceridemia, and low HDL in the Southern Chinese Han population [39]. A meta-analyses of 20 studies with 15,591 participants found that APOC3 Sst I and *rs2854116* polymorphisms might be associated with the risk of CAD [40]. Ding Y. et al*.*(2012) indicated that the minor alleles of *APOC3* -455 T/C polymorphisms were closely associated with acute coronary syndrome, which is a severe type of CAD, and the C allele was associated with higher TG and lower HDL cholesterol [41]. Thus, the *APOC3* might contribute to an increased risk of CAD as a result of its effect on lipid metabolism. Polymorphic variants prevent insulin binding, promoting the transcription and the synthesis of APOC3. As a result, the level of circulating APOC3 increases and acts as alipoprotein lipase inhibitor, leading to decreased clearance of TG-rich particles, which ultimately result in hypertriglyceridemia [42, 43]. The circulating TG-rich particles are preferentially taken up by the liver by means of a receptor-mediated process [44–46], which results in NAFLD and hepatic IR. Whereas a study in the Chinese Han population suggested that the two genetic variants (T -455C at *rs2854116* and C-482 T at *rs2854117*) in the *APOC3* were not associated with NAFLD risk, even did not contribute to the inter-individual differences in lipid profiles, insulin resistance, obesity, oxidative stress and susceptibility to NAFLD [47]. A case–control study found that the *APOC3* 3238G allele was significantly associated with increasing plasma TG levels and VLDL-C levels and enhanced risk of CAD via lipid metabolism [48].

## Peroxisome proliferator-activated receptors gene (*PPAR*)

There are three members of the PPAR family each encoded by a different gene: *PPAR* (*NR1C1*), *PPAR* (*NR1C3*), and *PPAR* (*NP1C2*). Carriers of the *PPARγ* Ala allele showed increased resistance to the development and progression of NAFLD by resisting oxidative stress [49, 50]. A Meta-analysis demonstrated a protective role for the Ala allele of the *PPARγ* Pro12Ala (*rs1801282*) polymorphism in NAFLD risk [51], and *rs1801282* polymorphism is associated with susceptibility to NAFLD in East Asians, but not in European populations [51]. Domenici F. A. et al*.* (2013) showed that 12 Ala allele of *PPARγ* was less prevalent among NASH patients than the healthy volunteers group. There were no associations among *PPARγ* SNPs (*rs1801282*) and clinical, laboratorial and histological parameters in NAFLD patients, suggest that the SNP *rs1801282* may result in protection against liver injury [52]. Leu162Val (*rs1800206*) *PPARα* SNP may be involved in the progression of NAFLD as the carriers have more advanced fibrosis [52]. While Wang J. et al*.* (2013) suggested that *rs1801282* polymorphism of *PPARγ* was not associated with NAFLD risk in both Asian and Caucasian descents from a meta-analysis [53]. A study reported that rs1800206 in *PPARα* was significantly associated with Lipoprotein (a) (Lp(a)) which is a LDL-like particle that can risk atherosclerosis independently and thus is the risk factor of CAD. And provide an evidence *PPAR α/γ* may influence the risk of dyslipidemia and cardiovascular diseases (CVD) via Lp (a) [54, 55]. The Ala 12 Ala genotype of the *PPARγ2* may decrease the number of diseased vessels and the severity of CAD, which could be because of a direct anti-atherogenic effect of this polymorphism as well as an indirect effect through its association with a lower level of inflammatory parameters and IR [56]. SNP *rs3856806* (also termed C161T or C1431T) in the *PPARγ* was significantly associated with fasted serum lipid profile in individuals with angiographically defined CAD since accumulating data support the role of *PPARγ* polymorphisms in CAD [57]. Wan J. et al. (2010) indicate that the *PPARγ rs3856806* polymorphism may reduce the risk of severe atherogenesis by modulation of adipose metabolism in Chinese patients with CAD [58]. We have previously demonstrated that the SNP *rs3856806* was associated with a higher susceptibility to NAFLD through the adiponectin pathway [59, 60].

## Sterol regulatory elementbinding proteins (SREBPs) / sterol regulatory elementbinding transcription factors (SREBFs) Gene

Three isoforms of SREBPs are encoded by two genes, *SREBP-1* (*SREBF-1*) and *SREBP-2* (*SREBF-2*). SREBP-1 target genes are involved in cholesterol biosynthesis, unsaturated fatty acid biosynthesis, triglyceride biosynthesis, phospholipid synthesis and lipid uptake [61, 62]. The *SREBF-1c rs11868035* polymorphism is associated with increased risk of developing NAFLD with more severe liver histology and derangement in glucose and lipoprotein metabolism, which contribute to the presentation and natural history of NAFLD [63]. SREBP-1c modulates the genetic susceptibility to the whole spectrum of health-related risk in NAFLD by extensively affecting multiple metabolic steps at hepatic and extra-hepatic sites. The SNP *rs11868035*, which is associated with impaired glucose homeostasis and lipoprotein and adiponectin responses to fat ingestion, was correlated with severity of steatosis and necro-inflammation and the presence of NASH [63]. And associated with CAD risk by modulating the changes in endothelial adhesion molecules [64, 65]. SREBP-2 encoded by a separate gene on human chromosome 22q13, which plays an important role in the maintenance of lipid homeostasis by stimulating the expression of genes correlated with the cholesterol biosynthetic pathways [66, 67]. Activation of the *SREBP-2* may play a critical role in enhancing cholesterol uptake and biosynthesis, and can be directly involved in the regulation of cholesterol metabolism in cells, thereby maintaining cholesterol homeostasis [68]. In recent years, some studies have shown that genetic polymorphisms of *SREBP-2* have a significant effect on the development of fatty liver disease, IR, and may result in hypertriglyceridemia [69, 70]. A case–control study in a Han Chinese population provided evidence that the GG genotype and G carrier (CG + GG) of *rs2228314* G > C polymorphism in *SREBP-2* may increase the risk of NAFLD. Thus, *SREBP-2 rs2228314* G > C polymorphism may be a potential biomarker for NAFLD [71]. A study from Eastern China manifests that *rs2228314* has no association with the risk of premature CAD nor extent of coronary lesions [72]. Further studies are required in this field. The C allele and the G/C genotype of *SREBP-2 rs2228314* were associated with increased risk of NAFLD in Asian Indians. As the G/C genotype of *SREBP-2* is significantly associated with Serum levels of TG, elevated CRP, fasting insulin and homeostasis model assessment for insulin resistance levels, it can be a potential explanation for the close relationship between IR, TG, CRP and *SREBP-2* polymorphism [73]. The functional SNP *rs133291* C/T in the *SREBF-2* gene has been linked to serum LDL cholesterol [74]. *SREBF-1c* SNPs and *SREBF-2* SNPs predicted the 7-year incidence of NAFLD and diabetes and endothelial dysfunction markers at the end of follow-up in nonobese, nondiabetic, insulin-sensitive subjects without metabolic syndrome at baseline. In biopsy-proven NAFLD patients, SREBF-2 predicted the presence of NASH and extensively affected tissue insulin sensitivity, pancreatic β-cell function, and lipoprotein and adipokine responses to fat ingestion. The *SREBF-1c* SNP, which is associated with impaired glucose homeostasis and lipoprotein and adiponectin responses to fat ingestion, was correlated with the severity of steatosis and necro-inflammation and the presence of NASH [63, 64].

## Transmembrane 6 superfamily member 2 gene (*TM6SF2*)

*TM6SF2* (*rs58542926* c.449 C > T) at the 19 p13.11 locus was associated with hepatic steatosis individuals genotyped using a human exome chip [75]. A exome-wide association studies identified the *rs58542926* C > T genetic variant of the *TM6SF2* gene, which encodes the E167K amino acidic substitution, as a determinant of hepatic TG content, serum aminotransferases, and lower serum lipoproteins [75, 76]. A case–control study in a community-based Han Chinese population highlight a missense variant in *TM6SF2* rs58542926 is significantly contributes to increased NAFLD risk in Chinese population, independent of the Patatin-like phospholipase domain-containing 3 gene *rs738409* and neurocan gene *rs2228603* polymorphisms, but whether it is also involved in NAFLD disease progression and severity needed further investigation [77]. Dongiovanni P. et al*.* (2015) suggested that carriers of E167K had a lower prevalence of plaques which is associated with risk factors for atherosclerosis, this protective effect of E167K against carotid atherosclerosis remained significant after adjustment for risk factors and NASH. The carriers have lower serum, and the inhibition of VLDL secretion from the liver protects against CVD, but at the cost of an increased risk of severe liver disease [78]. A mata-analysis showed that although *TM6SF2 rs58542926* T allele confers protection against CVD at the expense of higher risk for NAFLD, it does not explain the link between these two complex diseases [79].

## Microsomal triglyceride transfer proteingene (MTP/MTTP)

Act as a key role in lipid metabolism, MTP’s functional polymorphisms have previously been investigated. A recent meta-analysis indicates that *MTP* -493G > T (*rs1800591*) polymorphisms may contribute to the susceptibility to NAFLD patients. While *MTP rs1800591* polymorphism may be a valuable and practical biomarker for early detection of NAFLD [80]. In the Japanese population, a study showed that the-493 G-allele frequency was significantly higher in patients with biopsy-proven NASH than in the healthy control group, and the GG genotype in NASH patients predicted more severe steatosis [81]. However, the results regarding it are inconsistent. A study in a Brazilian population showed that there was no significant association between the *rs1800591* polymorphism and NAFLD [82]. Peng X. E. et al. (2014) showed that *MTP rs1800804* (−164 T/C) was associated with an increased risk of NAFLD and the level of TG was significantly lower in controls with the *rs1800804* (−164 C) risk allele [83]. *MTP* rs1800804 functional polymorphism and total cholesterol levels have an interaction. Thereby risk allele carriers with low cholesterol levels may be predisposed to an increased risk of developing CVD, which seems to be abolished among risk allele carriers with high cholesterol levels.

## Tumor necrosis factors-alpha gene (*TNF-α*)

Human TNF-α gene is located on chromosome 6p21.3. The protein it codes acts as a biomarker of systemic inflammation which contributes to IR via multiple direct endocrine and indirect modulatory effects of the genes involved in glucose and lipid metabolism [84, 85], and promotes inflammatory response to injury and regulates IR through insulin signal transduction pathways in the liver [86]. Previous studies have shown the association between the *TNF-α* polymorphisms and the risk of NAFLD, especially in Chinese origin patients. G/A variant at the *TNF-α* -238 increased susceptibility to NAFLD [60]. A study showed that *TNF-α* G to A polymorphism at position −238 was significantly associated with CAD and that the −238 A allele carriers exhibited an increased risk of developing CAD in Koreans, and therefore this allele could be used as a predictor for CAD in Koreans [87]. However, different ethnicity, phenotype, environment and other factors may have different results [88–91]. Variant at *TNF-α* -308 was notrelevant to NAFLD [60]. However, Hussain S. et al*.* (2015) showed that *TNF-α* -308 G > A polymorphism is significantly associated with CAD in the Pakistani population [92]. Recently, Chinese researchers provided preliminary evidence support the association between presence of a *TNF-α* -238 polymorphism and the developing of CAD in NAFLD patients of Chinese Han origin. They suggested that *TNF-α* -238 GA genotype may increase the risk for CAD in NAFLD patients. *TNF-α* -308 GA heterozygote genotype was positively associated with elevated levels of TG in NAFLD patients with CAD. Then obtained the conclusion that *TNF-α* -308 GA genotype plays a crucial role in the development of CAD in NAFLD patients [93].

## Manganese superoxide dismutase gene (*MnSOD*/*SOD2*)

Manganese superoxide dismutase (MnSOD) is an important antioxidant enzyme. There are data indicate that low MnSOD in rodent models of NAFLD may contribute to increased oxidative stress, other studies describe raised levels of this enzyme in NAFLD [94–96]. A case–control study and a meta-analysis highlight the mutant genotypes of TC + CC of C47T (*rs4880*) polymorphism in *SOD2* had a significant effect on the reduced of CAD risk [97]. Takami Y. et al*.* (2010) showed that systemic MnSOD protein is significantly higher in NASH patients than in steatotic patients and healthy controls [98]. A case–control and intra-familial allele association studies prove the evidence that carriage of the *rs4880* polymorphism involved in determining the susceptibility to oxidative stress and fibrogenesis, respectively, have been associated with the severity of liver fibrosis in adults and children with NAFLD [99].

## Conclusion

NAFLD and CAD has been a major public health problem affect millions of individuals worldwide. These two diseases are both recognized as the consequence of a complex interplay between genetics, diet and environment although a full understanding of pathogenesis has not yet been elucidated. Recent studies on the genetic polymorphisms association between NAFLD and CAD may provide a new prospective to the diagnosis, prevention and treatment of these two diseases. The role of genetic screening for NAFLD and CAD, while on the cusp, is still premature, further research are needed to exploring the mechanisms underlying this. In future, treatment would be more individualized and approaching to precision medicine depending on the reveal of these underlying mechanisms.
